# Over-Expression of Chorismate Mutase Enhances the Accumulation of Salicylic Acid, Lignin, and Antioxidants in Response to the White-Backed Planthopper in Rice Plants

**DOI:** 10.3390/antiox10111680

**Published:** 2021-10-25

**Authors:** Rahmatullah Jan, Muhammad Aaqil Khan, Sajjad Asaf, Lubna, In-Jung Lee, Kyung-Min Kim

**Affiliations:** 1 Division of Plant Biosciences, School of Applied Biosciences, College of Agriculture & Life Science, Kyungpook National University, 80 Dahak-ro, Buk-gu, Daegu 41566, Korea; rahmat2021@knu.ac.kr (R.J.); aqil_bacha@knu.ac.kr (M.A.K.); ijlee@knu.ac.kr (I.-J.L.); 2 Costal Agriculture Research Institute, Kyungpook National University, 80 Dahak-ro, Buk-gu, Daegu 41566, Korea; 3 Natural and Medical Science Research Center, University of Nizwa, Nizwa 616, Oman; sajjadasaf@unizwa.edu.om; 4 Department of Botany, Garden Campus, Abdul Wali Khan University, Mardan 23200, Pakistan; lubnabilal68@gmail.com

**Keywords:** lignin, antioxidant, signaling, relative expression, phenylalanine, proline

## Abstract

The white-backed planthopper (WBPH) is a serious pest of rice crop and causes sever yield loss each year, especially in Asian countries. In this study, we used *chorismate mutase* (*CM*) transgenic line to examine the defense mechanism of rice plants against WBPH. The survival rate of WBPHs, infestation rate of plants, lignin biosynthesis, transcriptional regulation of related genes, salicylic acid (SA) accumulation and signaling and antioxidants regulation were investigated. The WBPH population decreased by 67% in OxCM-t, and the plant infestation rate was 3.5-fold higher in wild-type plants compared with transgenic plants. A substantial increase in lignin was found in the transgenic line (742%) and wild-type (417%) plants. Additionally, *CM*, *phenylalanine ammonia lyase* (*PAL*), *chalcone synthase* (*CHS*), and *chalcone isomerase* (*CHI*) showed significant increases in their relative expression level in the transgenic line. Salicylic acid was significantly enhanced in the transgenic line compared with WBPH infestation. SA can activate *pathogenesis related proteins-1* (*PR1*), *PR2*, antioxidants, and the expression of their related genes: *superoxide dismutase* (*SOD*) and *catalase* (*CAT*). WBPH infestation reduced the chlorophyll contents of both transgenic and wild-type plants, but the reduction was great in wild-type than transgenic plants. The sugar content was only significantly increased in the transgenic line, indicating that sugars are not heavily involved in WBPH stress. Phenylalanine, proline, aspartic acid, and total amino acids were increased in the transgenic line and reduced in the wild-type plants. Taken together, all the results suggest that overexpression of *CM* gene regulates the defense mechanisms and enhances the rice toward WBPH stress.

## 1. Introduction

Rice is an essential staple food, and its production is constrained due to various environmental factors. The white-backed planthopper (WBPH), *Sogatella furcifera* (Horvath), is one of the most prominent causes of declining rice yields. It causes direct damage by sucking phloem sap and reduces phloem nutrients, which results in stunting, a decreased leaf area, chlorophyll contents, and photosynthetic rate, fewer tillers, loss of grain weight, and sometimes severe damage causing plant wilt and finally death [1]. Sometimes, nymph and adult WBPHs inject toxic saliva into the phloem causing hopper burn, which spreads throughout the plant, and in severe cases, plants die in substantial numbers, reducing yields substantially [2]. The response of plants to herbivores is associated with the mode of feeding and the area of infestation at the damaged site. Unlike chewing insects, phloem-feeding insects survive on a nutritionally imbalanced diet of phloem sap, which minimizes wound responses in host plants [3]. Generally, the exposure of plants to pathogens enhances the induction of genes involved in the shikimate pathway and aromatic amino acids (AAAs), such as phenylalanine (Phe), tyrosine (Tyr), and tryptophan (Trp). Pathogenic attack stimulates the plant cell wall and releases oligogalacturonides, which in turn stimulate the expression of various genes encoding enzymes of the shikimate pathway and AAAs as well as those encoding secondary metabolites derived from Phe and Tyr. Bacterial pathogens redirect normal host metabolism by delivering a constellation of type III effector proteins to enhance pathogen multiplication and nutrition. However, genes associated with AAAs have been significantly modified to cope with bacterial challenges, including cell wall alteration to control the nutrients and water passage from plants to invading bacteria. Phe, Tyr, and Trp are the main AAA molecules in plant metabolism, which are responsible for synthesis of a number of hormones, such as salicylic acid and auxin, as well as for essential secondary metabolites with many biological functions.

After insects feed on a plant, the host plant alters its defense mechanisms at transcriptomic, metabolomic, and hormonal levels. In response to insect infestation, the rice immune system and defense-related gene expression mitigates stress and protects the plants from huge devastation [4]. The innate immune system of rice is a complex system stimulated by various elicitors. The infestation of herbivores activates a plant defense system called herbivore-associated molecular patterns (HAMPs) [5]. During herbivory, plants recognize HAMPs and damage-associated molecular patterns (DAMPs) and activate various signaling pathways mediated by mitogen-activated protein kinase, jasmonic acid (JA) and salicylic acid (SA) [6]. SA and JA stimulate plant defense against pathogenic stress and regulate growth, and physiological, morphological, and biochemical processes. Many studies have reported that aphid infestation conspicuously regulates SA-related genes, whereas it reduces JA-related defense responses [7,8,9]. For instance, in *Arabidopsis*, silver leaf whitefly infestation induced SA and reduced JA defenses [10]. SA is well known as a key signal, which regulates local and systemic plant defense systems in response to pathogens attack [11,12]. In the SA signaling pathway, SA binding protein is considered to be the receptor of SA, which stimulate *pathogenesis-related* (PR) proteins and trigger systemic acquired resistance (SAR). Chorismate is essential in the synthesis of SA, as it is synthesized by two distinct pathways that are initiated by chorismate. One pathway is derived from phenylalanine, which is derived from chorismate by conversion to prephenate and then to phenylalanine [13]. In the second pathway, SA is produced through the conversion of chorismate into isochorismate and then to SA [14].

Rice responds to WBPH through the activation of oxidative and phenylpropanoid pathway enzymes, such as *chorismate mutase* (*CM*), *phenylalanine ammonia lyase* (*PAL*), *chalcone synthase* (*CHS*), *superoxide dismutase* (*SOD*), and *catalase* (*CAT*) [15], which enhance lignification in cell walls and increase toughness of the cell wall. Brown planthopper (BPH) infestation stimulates antioxidant-related genes, such as *SOD* and *CAT*, in rice [15]. The *SOD* gene encodes superoxide dismutase enzyme, which catalyzes the superoxide radical into H_2_O_2_ and the *CAT* gene encodes the catalase enzyme, which catalyzes the conversion of H_2_O_2_ into H_2_O and O_2_ and provides plant protection against oxidative stress. Lignin is a natural polymer embedded in cell walls, and it provides rigidity and plays a key role in mechanical support and defense mechanisms. Depending on the situation, lignin can play protective, disruptive, and sustaining roles in plant stress conditions [16]. Lignin is a defensive barrier against pathogens and herbivores due to its complex structure. A study conducted on *Gossypium barbadense* showed that lignin biosynthesis enhanced tolerance against *Verticilium dahlia* and PAL pathway genes were upregulated [17]. Mutant Arabidopsis line of PAL gene showed reduced lignin and SA contents as compared to wild-type, which also showed enhanced susceptibility to pathogens [14]. In response to pathogen infestation, SA stimulates PR proteins, which accumulate more in the infected tissues and can protect the plant from further damage [18]. The reduction or stopping of SA accumulation can compromise plant immunity [19]. *PR1* and *PR2* are induced in response to rice strip virus and BPH [20]. SA signaling activates PR and oxidative responsive genes, which subsequently increase the detoxification of reactive oxygen species (ROS). PR genes encode protease inhibitor proteins, which are pathogenic wound responsive proteins, and they suppress digestion in some chewing insects and nematodes in their midguts [21,22]. SA is required for defense against insects and pathogens, but the current study was conducted to clarify the regulatory behavior of SA and induction of PR genes with the over-expression of the chorismate mutase (*CM*) gene. Further, the study aimed to determine whether the over-expression of the *CM* gene increases resistance to WBPH infestation through lignification and the stimulation of oxidative responsive apparatus mediated by SA.

## 2. Materials and Methods

### 2.1. Plant Material, Growth Conditions and Phenotypic Evaluation

In the current experiment, we used TN1 rice plants and *CM* transgenic [23] and non-transgenic Cheongcheong rice plants Kindly provided by Plant Molecular Breeding Lab, Kyungpook National University, Korea. The seeds were treated with fungicides overnight and then washed three times with double-distilled water. Then, the clean seeds were soaked for three days in water at 32 °C in the dark, and the water was changed every day following Jan et al. [24]. During the incubation period, the seeds were sprouted and transferred to autoclaved soil and kept in the dark again for three days. After successful growth, the seedlings were exposed to light and kept in a greenhouse for further experimentation.

### 2.2. Experimental Design

We selected three groups of Cheongcheong plants to evaluate the effect of WBPH on rice plants. The first group was selected as the control group, which was the wild-type group, and it was not infected with WBPH (Wt-cont). The second group was also wild-type, but it was infected with WBPH and given the name wild-type-treated (Wt-t). The third group was *CM* over-expressor (OxCM), and it was infected with WBPH and given the name OxCM-treated (OxCM-t). The WBPH population was obtained from the Rural Development Administration center in Jeongu, Korea. The plant hoppers were reared in a transparent glass insectarium, with a 50 cm × 50 cm × 40 cm length, width, and height, respectively, throughout the year, and they were fed with two-week-old seedlings of susceptible TN1 rice cultivars. Due to the short lifespan of adult WBPHs, we used third-instar nymphs to emerging adult insects to conduct tests. We grew 100 plants of each group in a single seedling box and kept them separately in an insectarium to evaluate the effect of WBPHs and the tolerance of the OxCM line toward the insect. After the 3rd to 4th leaf stage, each Wt-t and OxCM-t plant group was infected with 100 (40 male, 60 female) WBPHs, which were starved two hours before infestation. Further, data were collecting according to each section of the experiment. Data for the insect survival assessment were collected after 5, 10, and 15 days of infestation from Wt-t and OxCM-t plants. The number of newly hatched instar were counted after 20, 23, 26, and 29 days of infestation, while the rates of infected and non-infected plants were collected after 3, 6, 9, 12, and 15 days of infestation. Plants were considered infected when they showed the first symptom of infestation. Further data were collected according to further required experimentation.

### 2.3. RNA Isolation and qRT-PCR

We collected leaf samples for total RNA extraction from the plants of all three groups in triplicates after 0, 1, 6, 12, 24, and 36 h of WBPH infestation to check the selected gene expression level. RNA extraction, cDNA synthesis, and qRT-PCR were performed following Jan et al. [24]. The primer and accession number for each gene is listed in Table 1. We used RNeasy Plant Mini Kits (50) Qiagen (Hilden, Germany), for the total RNA extraction and qPCRBIO and qPCRBIO SYBR Green kit (PCR Biosystems) for cDNA synthesis and qRT-PCR, respectively. Actin was used as a reference gene. An Eco Real-Time (Illumina, Singapore) machine was used for qRT-PCR reaction using 20 µL (10 µL SYBR green, 7 µL ddH_2_O, 1 µL template DNA, and 1 µL primer) of reaction volume.

### 2.4. Measurement of Lignin Content

We followed a modified version of a previously reported method [25] to quantify the lignin content of the leaves. Fresh leaf samples were collected in triplicate from the control, Wt-t, and OxCM-t plants after 0, 3, 6, 12, and 24 h of WBPH infestation. A measure of 1 g of each sample was ground into fine powder in liquid nitrogen and washed with 95% ethanol to remove soluble compounds. The remaining sample was further washed with pure acetone and dried completely in an incubator. After drying, the sample was further treated with acetic acid and sonicated with an ultrasonic machine and then centrifuged at 3000× *g* for 5 min. Then, the supernatant was removed, and the pellet was resuspended in 25% acetyl bromide and centrifuged again at 3000× *g* for 5 min. Further, the sample was mixed with a mixture of acetic acid and acetyl bromide (4:1, *v/v*) and heated at 70 °C for 2 h. Then, the sample was cooled down at room temperature (25 °C) and transferred into a 50 mL tube containing 2 M sodium hydroxide and 7.5 M hydroxylamine hydrochloride. Acetic acid was used to equalize the volume of each of the samples. The absorbance of each sample was measured using a spectrophotometer at 280 nm (UV-2450; Shimadzu, Japan), and the lignin contents were measured as OD_280_ mg/g fresh weight. 

### 2.5. Quantification of Endogenous Salicylic Acid

We quantified SA accumulation sin response to WBPH. The leaves from the control, Wt-t, and OxCM-t plants were collected after 0, 3, 6, 12, and 24 h, and the freeze-dried samples were ground in liquid nitrogen into a fine powder. A sample of 0.3 g of the powder was homogenized with 90% ethanol and 100% methanol and centrifuged for 20 min at 1000 rpm. Then, the supernatant was collected, and the methanol of the supernatant was dried in a vacuum centrifuge. Next, it was resuspended in 5% trichloroacetic acid (3 mL). The supernatant was further mixed with ethyl acetate/cyclopentane/isopropanol (49.5:49.5:1, *v/v*), and the uppermost organic layer was collected in a 4 mL vial and dried with nitrogen gas. The extracted SA was analyzed using high-performance liquid chromatography (HPLC) and quantified using fluorescence detection using a Zorbax Eclipse XDB-C18 column (Agilent Technologies, Santa Clara, CA, USA).

### 2.6. Histological Staining

The hypersensitive response (HR) of Wt-t and OxCM-t plants toward WBPH infestation was compared with that of control plants using trypan blue histological staining following Koch and Slusarenko [26]. Five leaves were randomly collected from control, Wt-t, and OxCM-t plants after 0, 3, 6, 9, and 12 days of WBPH infestation, and the HRs were determined visually. Cell death was quantified using the density of trypan blue in each leaf.

### 2.7. Antioxidant Enzyme Assay

Antioxidant enzymes, glutathione peroxidase (GPx), and lipid peroxidase (MDA = malondialdehyde), were measured using the Glutathione Peroxidase Cellular Activity Assay Kit (Sigma, Burlington, MA, USA) and Lipid Peroxidation (MDA) Assay Kit (Sigma, Burlington, MA, USA), respectively, following the manufacturer’s protocols. About 100 mg of fresh leaves were randomly collected after 0, 3, 6, 12, and 24 h of WBPH infestation to detect the activity of the two enzymes. Leaves for the detection of GPx activity were ground in liquid nitrogen, homogenized in 3 mL of 5% trichloroacetic acid (TCA), and centrifuged at 15,000 rpm for 15 min following Bilal et al. [27]. The supernatant was collected in a new 2 mL tube and used for further analysis. A sample, positive control, and blank reaction were performed according to the scheme shown in Table 2. According to the user manual, one vial of NADPH assay reagent was reconstituted in 1.25 mL of ddH_2_O. An amount of 30 mM of tert-butyl-hydroperoxide was prepared by dilution of 21.5 µL of Luperox TBH70X in 5 mL of ddH_2_O. Then, 250 µL of the prepared reaction described in Table 1 was placed in a 96-well microplate. The reaction was started by adding 10 µL of 30 mM tert-butyl hydroperoxide. A decline in absorbance at 340 nm was calculated using Shimadzu spectrometer (Shimadzu, Kyoto, Japan), with an initial delay of 15 s and interval of 10 s. Readings were taken six times. The absorbance was calculated in units/mL using the following formula:

ΔA_340_/6.22 × DF/V

where

ΔA_340_ = A_340_/min _(blank)_ − A_340_/min _(sample)_;
6.22 = Ɛ^mM^ for NADPH;
DF = dilution factor of sample before adding to reaction;
V = sample volume in mL.

For lipid peroxidation, the kit provided the MDA lysis buffer, phosphotungstic acid, BHT 100X, TBA, and MDA standard 4.17 M. The TBA solution was reconstituted by adding 7.5 mL of glacial acetic acid (not provided), the volume was adjusted to 25 mL by adding ddH_2_O, and the solution was sonicated. A 10 µL measure of 4.17 M MDA solution was diluted with 407 µL ddH_2_O to prepare 2 mM of standard MDA. Then, 100 µL of diluted solution was added to 900 µL of ddH_2_O to prepare 0.2 mM MDA solution. Subsequently, 0, 2, 4, 6, 8, and 10 µL of 0.2 mM MDA standard solution was added into a 96-well microplate and 0 (blank) 0.4, 0.8, 1.2, 1.6, and 2.0 µL standards were prepared. Thereafter, ddH_2_O was added to each tube to reach a volume of 200 µL. Samples were prepared by homogenizing 10 mg of tissue with 300 µL of MDA lysis buffer containing 3 µL of BHT on ice. The samples were centrifuged for 10 min at 13,000 rpm, and the residue was discarded. Then, 200 µL of each sample was placed in a 1 mL tube, to which 600 µL of TBA was added, incubated for 1 h at 95 °C and cooled by keeping it on ice for 10 min. Finally, 200 µL of the blank and samples were pipetted into a 96-well microplate and absorbance was analyzed at 532 nm on Shimadzu spectrometer (Shimadzu, Kyoto, Japan). The reaction was run in three technical replicates and data were calculated using the following formula:

S_a_/S_v_ × D = C (1)

S_a_ = amount of MDA in unknown sample (nmole);
S_v_ = sample volume added into each well (mL);
D = sample dilution factor;
C = concentration of MDA in the sample.

### 2.8. Amino Acid Isolation and Chlorophyll Content

Phenylalanine, tyrosine, proline, arginine, aspartic acid, and total amino acids were quantified following Jan et al. [28]. A total of 1 g of freeze-dried leaf sample was ground into a fine powder in liquid nitrogen and extracted with 20 mL of 70% HPLC grade methanol with shaking for 24 h. The amino acid contents were evaluated using the EZ fast analysis kit (Phenomenex, Santa Clara, CA, USA). The amino acid contents were determined using gas chromatography-mass spectrometry using a Hewlett-Packard (HP) 6890N/5975 instrument (Agilent Technologies, Torrance, CA, USA) and a ZB-aromatic amino acid (AAA) (10 m × 0.25 mm) amino acid analysis column, with constant carrier gas flow and an oven temperature program as described by Pavlík et al. [29]. The chlorophyll contents were measured using a soil plant analysis development chlorophyll meter using randomly selected leaves in triplicate after the 1st, 2nd, 3rd, 4th, and 5th day of WBPH infestation. Soluble sugar contents were quantified, following Jan et al. [28], using HPLC equipped with a Bio-Rad Aminex 87C column (300 × 7.8 mm), and water was used as an eluent with a flow rate of 0.6 mL/min.

## 3. Statistical Analysis

All experiments were performed in triplicate, and the data from each replicate were pooled. Data were analyzed using two-way analysis of variance (ANOVA), followed by the Bonferroni post hoc test. A completely randomized design was used to compare the mean values of different treatments. Data were graphically presented, and statistical analyses were performed using GraphPad Prism software (version 5.01; GraphPad, San Diego, CA, USA).

## 4. Results

### 4.1. Characterization of Symptoms and Survival Rate of White-Backed Planthopper

**Figure 1:**
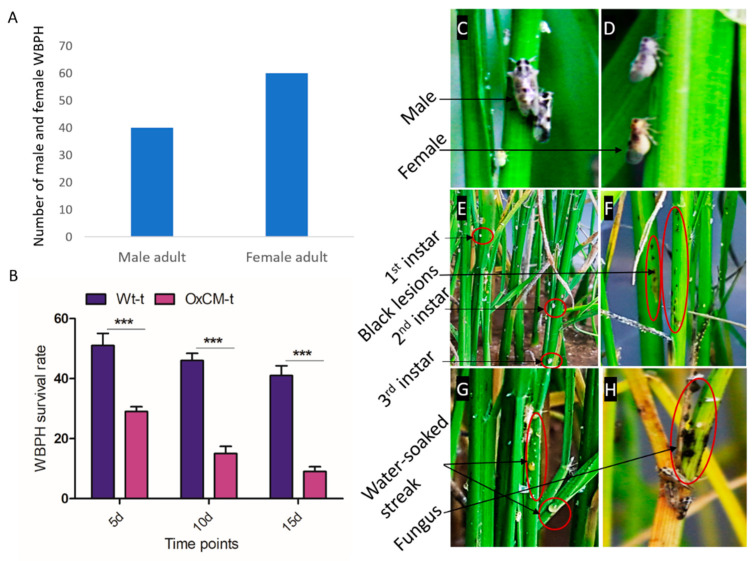
White-backed planthopper (WBPH) survival rate, their attack approach and symptoms on plant. (**A**) A total of 100 WBPH were infested out of them, 40 were male and 60 were female. (**B**) Rate of survival of WBPH on both wild-type plants infected with WBPH (Wt-t) and chorismate mutase transgenic over-expressor plants infected with WBPH (OxCM-t) after 5, 10, and 15 days of infestation. (**C**,**D**) Male and female respectively, feeding on seedling stem. (**E**) Identification of 1st, 2nd, and 3rd instar of WBPH life cycle. (**F**–**H**) Different symptoms of WBPH infestation, such as black lesion, green-yellow water streak, and fungal infestation respectively. Graph bars indicate the mean ± standard deviation (*n* = 3), and asterisks indicate significant differences (*** *p* < 0.001) analyzed using two-way analysis of variance and Bonferroni post hoc test. Data presented in graphical form are the means for three independent experiments.

We infected rice plants with 40 male and 60 female WBPHs to evaluate the effects of WBPH on rice plants (Figure 1A). The number of females was higher than the number of males due to the higher death rate of females than males. We evaluated the survival rate of WBPHs in Wt-t and OxCM-t plants and found that the WBPH survival rate was reduced by 15% after 5 d, 51% after 10 d, and 67% after 15 d of infestation in OxCM-t plants compared with Wt-t plants (Figure 1B). The exact reason for WBPH death is still unknown, but we assumed that the OxCM plants were more resistant to the WBPH than the wild-type. Throughout our experiment, we noticed that both male and female WBPHs mostly fed on the lower stem, and newly hatched instar were observed on the lower stem (Figure 1C–E). After 3–5 days of infestation, symptoms mostly appeared on the lower part of the stem. Different symptoms appeared depending on the duration of infestation. The initial symptoms appeared in the form of small dark dots (Figure 1F). After about 10 to 15 days of continuous infestation, another symptom appeared in the form of water streaks on the lower stem and midrib of the leaves (Figure 1G). After continuous infestation for about a month, the new instar hatched and the plants came under the severe threat, turning yellow and starting to die (Figure 1H). At this stage, a fugal attack also appeared, which shows that WBPH infestation may also transmit fungal disease, as some of the insects act as a vector for bacteria and fungi.

### 4.2. White-Backed Planthopper Population Growth and the Resistance of Chorismate Mutase Transgenic Over-Expressor Plants

**Figure 2:**
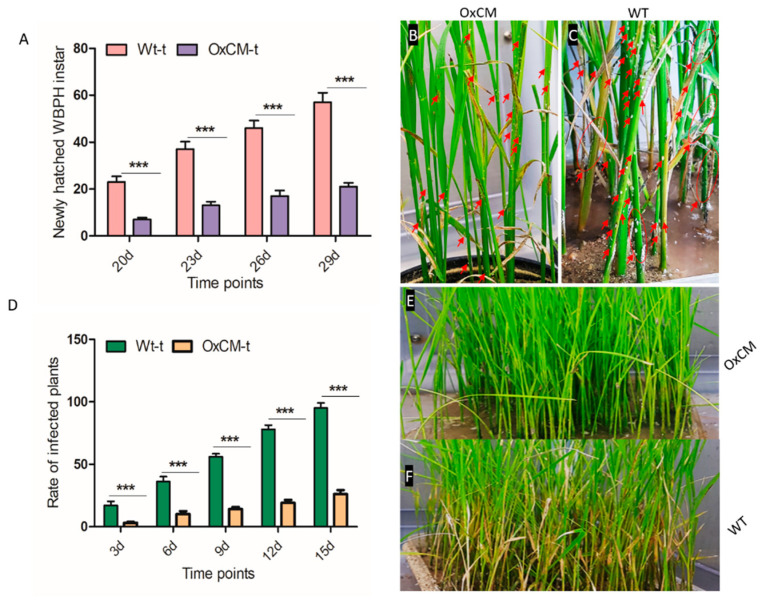
(**A**) Newly hatched instars of white-backed planthopper (WBPH) in wild-type plants infected with WBPH (Wt-t) and chorismate mutase transgenic over-expressor plants infected with WBPH (OxCM-t) after 20, 23, 26, and 29 days of infestation. (**B**,**C**) Population of newly hatched instar on OxCM and Wt plants, denoted by arrows. (**D**) WBPH infected plants of OxCM and Wt after 3, 6, 9, 12, and 15 days of infestation. (**E**,**F**) Pictorial representation of WBPH infestation of OxCM and Wt respectively. Graph bars indicate the mean ± standard deviation (*n* = 3), and asterisks indicate significant differences (*** *p* < 0.001) analyzed using two-way analysis of variance and Bonferroni post hoc test. Data presented in graphical form are the means for three independent experiments.

The population growth and survival rates of newly hatched instar WBPHs were determined. Usually, a single female WBPH laid about 119–158 eggs on the leaf midrib or stem in a cluster of 5–30 eggs, which were not visible to the naked eye [2]. After 17 to 20 days of infestation, new nymphal instars appeared on both Wt-t and OxCM-t plants. We counted the number of newly hatched instars after 20, 23, 26, and 29 days of infestation. We found that the WBPH population on Wt-t plants was approximately 2-fold higher than on OxCM-t plants after 20–29 days of infestation (Figure 2A). Figure 2B,C show few instars on OxCM-t plants, while Wt-t plants had substantial numbers of newly hatched instars, which are indicated by arrows, showing that OxCM plants are resistant to newly hatched WBPH and reduce the WBPH population. Further, we evaluated the rate of infestation of Wt-t and OxCM-t plants after 3, 6, 9, 12, and 15 days of infestation. The statistical analysis showed that the rate of infected plants increased substantially in Wt-t plants compared with OxCM-t plants (Figure 2D). The percentage of infected Wt-t plants increased by 4.5, 2.5, 3, 3, and 3.5 fold compared with OxCM-t plants after 3, 6, 9, 12, and 15 days of infestation, respectively. Figure 2E,F show the infestation pattern of OxCM-t and Wt-t plants, respectively, after WBPH infestation. These results showed the increased number of new instar on wild-type plants and high infection rate of wild-type plants indicated that wild-type plants were susceptible to WBPH, while transgenic plants showed enhanced tolerance toward WBPH.

### 4.3. Chorismate Mutase Enhances Lignin Contents through the Induction of Downstream Genes

**Figure 3:**
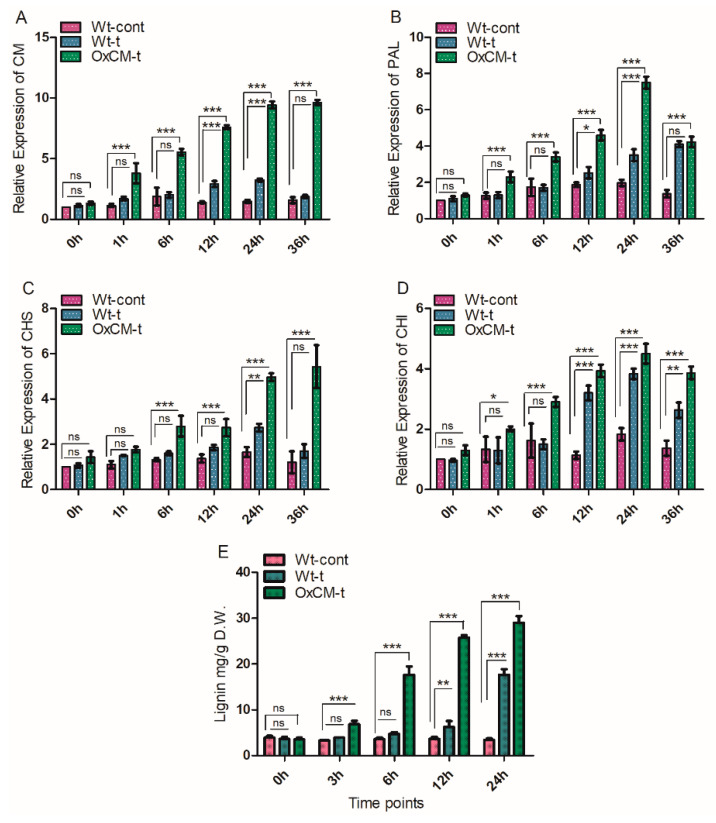
Over-expression of chorismate mutase (*CM*) gene positively regulates downstream genes and lignin accumulation. Relative transcriptional accumulation of (**A**) *CM* gene, (**B**) *PAL* gene, (**C**) *CHS* gene, and (**D**) *CHI* gene in the wild-type control (Wt-cont); wild-type infected with WBPH (Wt-t) and chorismate mutase transgenic over-expressor infected with WBPH (OxCM-t) plants in response to WBPH infestation. The relative expression of each gene was measured after 0, 1, 6, 12, 24, and 36 h of WBPH infestation. The actin gene was used as a reference gene. (**E**) Lignin quantification after 0, 3, 6, 12, and 24 h of infestation. Graph bars indicate the mean ± standard deviation (*n* = 3), ns indicates non significant and asterisks indicate significant differences (* *p* < 0.05, ** *p* < 0.01, *** *p* < 0.001) analyzed using two-way analysis of variance and Bonferroni post hoc test. The experiments were repeated three times.

Lignin is the second most abundant biopolymer, and it acts as an essential barrier for pests and pathogens [30]. It is produced in the plant cell by the phenylalanine pathway, and the entire reaction is catalyzed by about ten enzymes [31]. We evaluated the expression of *CM, PAL, CHS,* and *CHI* genes and quantified the accumulation of lignin contents in Wt-cont, Wt-t, and OxCM-t plants after 0, 1, 6, 12, 12, and 36 h of WBPH infestation, respectively (Figure 3). The CM gene was consistently expressed in the OxCM line due to the 35S promoter, while in the Wt-t line, expression was significantly increased after 12 and 24 h of infestation compared with Wt-cont plants (Figure 3A). The expression pattern of PAL was identical to the expression pattern of the CM gene in OxCM-t plants: it was expressed consistently and significantly from 1 to 24 h after WBPH infestation (Figure 3B). However, expression was reduced after 36 h of infestation. PAL was also more expressed in the Wt-t than Wt-cont plants. However, expression was not significantly different, except at 12 and 24 h, and it was less than the expression in OxCM-t plants (Figure 3B). *CHS* is another one of the prominent genes in the phenylpropanoid pathway, and it actively participates in lignin biosynthesis. The evaluation of the expression of *CHS* under WBPH infestation indicated that *CHS* was significantly induced in the OxCM-t plants after 6 h of WBPH infestation compared with the Wt-cont plants (Figure 3C). However, the expression of *CHS* in the Wt-t plants was significantly increased as compared with Wt-cont plants. *CHI* is another important gene that is part of the phenylpropanoid pathway and involved in the biosynthesis of lignin. Our study indicated that the expression of *CHI* was significantly higher in OxCM-t than Wt-cont plants after 1, 6, 12, 24, and 36 h of WBPH infestation (Figure 3D). The expression level of *CHI* was also higher in Wt-t than Wt-cont plants after 12, 24, and 36 h of WBPH infestation. Lignin quantitative analysis indicated that OxCM-t plants accumulated lignin at substantial quantities compared with both Wt-cont and Wt-t plants (Figure 3E). In OxCM-t plants, the accumulation of lignin increased by 109%, 388%, 598%, and 752% compared with Wt-cont plants after 3, 6, 12, and 24 h of infestation, respectively. However, in Wt-t plants, it increased by 9%, 30%, 68%, and 417% compared with Wt-cont plants after 3, 6, 12, and 24 h of infestation, respectively.

### 4.4. Salicylic Acid Stimulates PR1 and PR2 Genes in Response to White-Backed Planthopper Infestation

**Figure 4:**
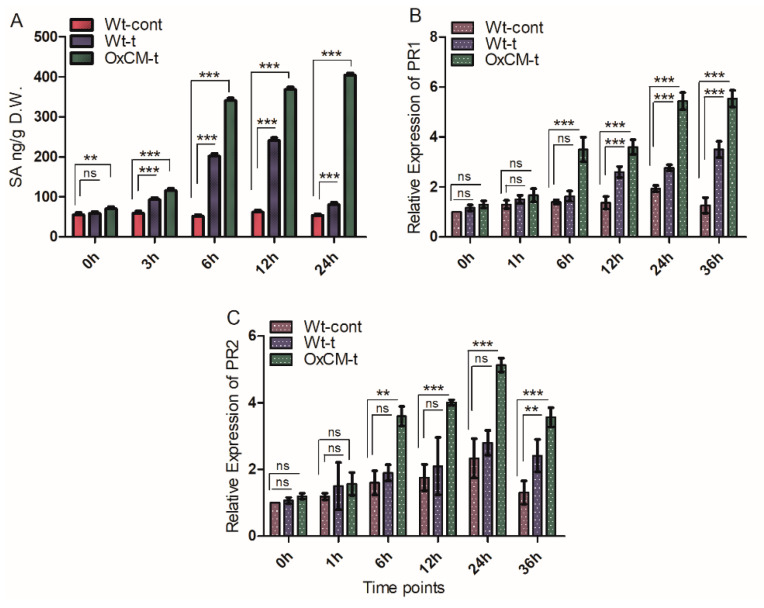
White-backed planthopper (WBPH) induces salicylic acid signaling and pathogenesis-related gene expression. (**A**) SA accumulation after different time points in wildtype control plants (Wt-cont), wild-type plants infected with WBPH (Wt-t), and chorismate mutase transgenic over-expressor plants infected with WBPH (OxCM-t). (**B**,**C**) Relative expression of *PR1* and *PR2* genes, respectively, at different timepoints after WBPH infestation. Actin was used as a reference gene. Graph bars indicate the mean ± standard deviation (*n* = 3), ns indicates non significant and asterisks indicate significant differences (** *p* < 0.01, *** *p* < 0.001) analyzed using two-way analysis of variance and Bonferroni post hoc test. The experiments were repeated three times.

SA is an important phytohormone, which is induced during biotrophic pathogenic attack, and its deployment is monitored via PR genes [32,33]. Therefore, we aimed to investigate the role of the *CM* gene in SA pathway signaling and to evaluate the transcriptional regulation of the PR1 and PR2 genes, which are the key marker genes associated with the SA pathway. We investigated the accumulation of SA in OxCM-t and Wt-t plants in comparison with Wt-cont plants under WBPH pathogenic stress. We found that SA was significantly higher in both Wt-t and OxCM-t plants than Wt-control plants, but the accumulation of SA in OxCM-t plants was higher than in the Wt-t plants (Figure 4A). The accumulation of SA in Wt-t plants increased by 5%, 57%, 296%, 288%, and 50% after 0, 3, 6, 12, and 24 h of infestation, respectively, compared with Wt-cont plants. However, the accumulation of SA increased by 27%, 94%, 568%, 493%, and 48% in OxCM-t plants after 0, 3, 6, 12, and 24 h of infestation, respectively, compared with Wt-t plants. This indicates that over-expression of the *CM* gene significantly increases the accumulation of SA associated with WBPH infestation. Initially, the expression of the *PR1* gene in Wt-t plants was not significantly different to that in Wt-cont plants. However, after 12, 24, and 36 h of infestation, *PR1* gene expression was significantly higher in Wt-t plants than Wt-cont plants (Figure 4B). Additionally, *PR1* expression was significantly regulated in OxCM after 6, 12, 24, and 36 h of infestation, and the expression level increased consistently compared with the Wt-cont plants (Figure 4B). Moreover, the *PR2* gene was also upregulated in a similar manner to *PR1* gene in the OxCM-t plants: after 6, 12, 24, and 36 h of infestation. However, the *PR2* gene in the Wt-t plants was not significantly upregulated compared with the Wt-cont plants until 36 h after infestation (Figure 4C). This transcriptional regulation pattern indicates that SA is responsible for the induction of the *PR1* and *PR2* genes during WBPH infestation. These results show that transgenic plants under WBPH infestation increase endogenous SA level as well as induce SA signaling. 

### 4.5. Hypersensitive Responses and Regulation of Antioxidant Apparatus

**Figure 5:**
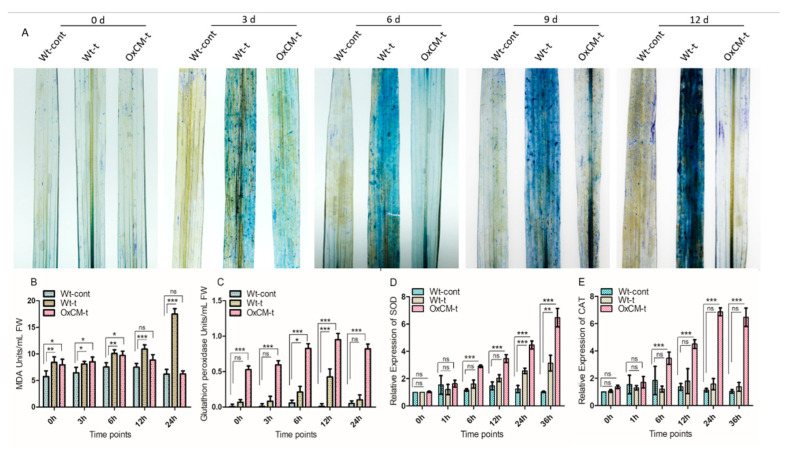
Over-expression of the chorismate mutase gene enhances resistance toward white-backed planthopper (WBPH) stress through the induction of hypersensitive responses and antioxidant machinery. (**A**) Induced hypersensitive responses after 0, 3, 6, 9, and 12 days of WBPH infestation. (**B**) Catalase and (**C**) glutathione peroxidase accumulation in response to WBPH infestation at different time points. (**D**,**E**) Relative transcriptional accumulation of *SOD* and *CAT* genes, respectively, against WBPH infestation. Actin was used as a reference gene. Graph bars indicate the mean ± standard deviation (*n* = 3), ns indicates non significant and asterisks indicate significant differences (* *p* < 0.05, ** *p* < 0.01, *** *p* < 0.001) analyzed using two-way analysis of variance and Bonferroni post hoc test. The experiments were repeated three times. Wt-cont; Wt-t, wild-type infected with WBPH; and OxCM-t, chorismate mutase transgenic over-expressor infected with WBPH.

The HR is an important defense system that is initiated at the site of infestation. As the HR, ROS, and antioxidants are interlinked in the defense system, we investigated the HR using quantitative visualization of tryptophan blue, we quantified lipid peroxidase (MDA, malondialdehyde) and glutathione peroxidase (GPx), and we evaluated the level of expression of the *SOD* and *CAT* genes (Figure 5). Leaf samples were collected randomly for the detection of cell death from Wt-cont, Wt-t, and OxCM-t plants after 0, 3, 6, 9, and 12 days of infestation. We found substantial cell death in Wt-t leaves after 9 and 12 days of infestation compared with Wt-cont and OxCM-t plants (Figure 5A). This extensive cell death in Wt-t plants shows that they are more susceptible to WBPH infestation. Furthermore, the antioxidant evaluation indicated that MDA was significantly (*p* < 0.001) enhanced consistently throughout the infestation in the Wt-t plants compared with Wt-cont plants (Figure 5B). However, MDA accumulation did not increase significantly (*p* < 0.05) in OxCM-t plants after 0, 3, and 6 h of WBPH infestation, while it reduced after 12 and 24 h of infestation. Unlike MDA, the GPx concentration was significantly (*p* < 0.001) and consistently enhanced in the OxCM-t plants compared with the Wt-cont plants after WBPH infestation (Figure 5C). The accumulation of GPx in Wt-t plants in response to WBPH stress was also significantly enhanced after 6 and 12 h of infestation compared with Wt-cont plants. The accumulation of MDA and GPx in the transgenic line indicated that MDA reduces and GPx increases with increasing WBPH infestation. We investigated the transcriptional level *SOD* and *CAT* gene in Wt-cont, Wt-t, and OxCM-t plants after 0, 3, 6, 12, 24, and 36 h of WBPH infestation to further evaluate the antioxidant apparatus in response to WBPHs (Figure 5D,E). The quantitative transcription of *SOD* and *CAT* showed the same pattern of expression: they were enhanced consistently and significantly (*p* > 0.001) after 6, 12, 24, and 36 h of WBPH infestation compared with Wt-cont plants. Additionally, there was a significant (*p* < 0.001) increase in *SOD* in Wt-t plants after 24 h of infestation compared with Wt-cont plants (Figure 5D), while irregular expression of *CAT* was found in Wt-t plants throughout the infestation period (Figure 5E). The transcriptional regulation of both the *SOD* and *CAT* genes indicated that the *CM* gene is associated with the regulation of antioxidant-related genes.

### 4.6. White-Backed Planthoppers Alter Chlorophyll, Sugar and Amino Acid Contents

**Figure 6:**
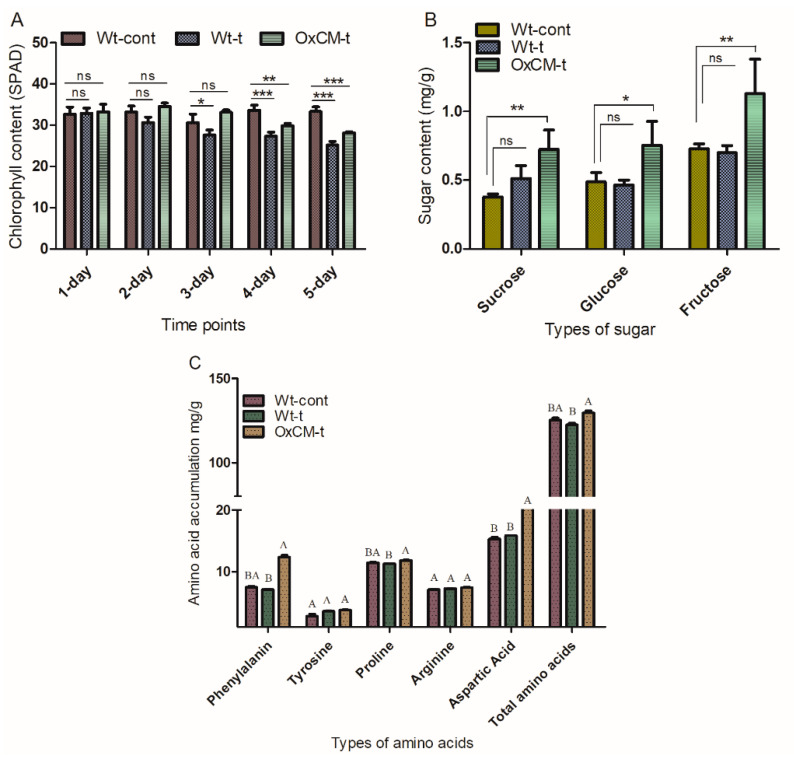
WBPH infection regulates chlorophyll, sugar and amino acid contents. (**A**) Inhibition of chlorophyll contents in Wt-cont, Wt-t and OxCM-t plants measured by SPAD. (**B**) Sucrose, glucose and fructose accumulation in Wt-cont, Wt-t and OxCM-t plants under WBPH infestation. (**C**) Various free amino acid and total amino acid quantification under WBPH infestation. Graph bars in (**A**,**B**) indicates mean ± standard deviation (*n* = 3), ns indicates non significant and asterisks shows a significant difference (* *p* < 0.05, ** *p* < 0.01, *** *p* < 0.001) analyzed by two-way ANOVA, Bonferroni post hoc test. Different letters in (**C**) represents significant differences between means analyzed by Duncan’s multiple range test (DMRT). The experiments were repeated three times.

We compared the chlorophyll, sugar, and amino acid contents of Wt-t and OxCM-t plants with Wt-control plants. The chlorophyll contents were investigated after 1, 2, 3, 4, and 5 days of infestation, and we found significant (*p* < 0.001) decreases in both Wt-t and OxCM-t plants after 4 and 5 days compared with Wt-cont plants (Figure 6A). Initially, the chlorophyll contents were increased in the OxCM-t plants compared with the Wt-control. However, after 4 and 5 d, the chlorophyll contents were reduced. However, throughout the infestation, the chlorophyll concentration in the OxCM-t plants was higher than in the Wt-t plants. Sucrose, glucose, and fructose were also significantly enhanced (*p* < 0.001) in OxCM-t plants compared with Wt-t plants (Figure 6B). Furthermore, we investigated the phenylalanine, tyrosine, proline, arginine, aspartic acid, and total amino acid concentrations in all three groups of plants (Figure 6C). We did not find any significant difference in tyrosine and arginine, while phenylalanine, proline, aspartic acid, and total amino acids were significantly enhanced in OxCM-t plants compared with Wt-cont plants. In contrast to OxCM-t plants, phenylalanine, proline, and total amino acids were significantly reduced in Wt-t plants compared with Wt-cont plants, which indicates that phenylalanine, proline, and total amino acids could be directly or indirectly involved in the mitigation of WBPH infestation. 

## 5. Discussion

Host plant resistance provides evidence of effective, environmentally friendly, and economical techniques for dealing with crop pests [34,35]. Currently, rice planthoppers, primarily the WBPH, are the most devastating insect pests, and they cause severe damage to plants by direct feeding or indirect disease transmission, which leads to severe yield losses [36,37]. Previously, we identified and mapped the *CM* gene, which is resistant to brown planthopper (BPH), using QTL analysis and developed a transgenic rice line [38]. In the current study, we characterized the *CM* gene to establish prolonged resistance of the rice cultivar against WBPH and to contribute to sustainable rice production.

WBPHs activate the plant defense mechanism including, PR genes, metabolomic induction and signaling cascades. Under pathogenic attack, plant defense regulators against pathogen infestation are induced or suppressed, and their interplay indicates the level of resistance needed for the plant-triggered immunity system. Oligogalacturonide and several transcription factors (TFs) induced by pathogenic attack are responsible for the regulation of genes associated with shikimate and AAAs biosynthesis pathways and the genes responsible for the synthesis of secondary metabolites from the AAAs [39]. In *Arabidopsis*, *AtMYB15* induces all the genes in the shikimate pathway, which are also induced by wounding [40]. Our data show that *CM*, *PAL*, *CHS*, and *CHI* were significantly upregulated in the OxCM-t line after WBPH infestation (Figure 3). Although the *CM* gene was consistently expressed in the OxCM-t line due to the S35 promoter, however the expression of *CM* was also increased significantly in the Wt-t plants compared with the Wt-cont plants after 12 and 24 h of WBPH infestation. This phenomenon indicates that WBPH induces *CM* gene expression in rice plants. It has been reported that the elicitor-treated plant tissues showed strong expression of *CM*, *PAL*, *CHS*, and *CHI* compared with control plants [41], which indicates that these genes are expressed collectively in response to stress conditions. Our results show that *CM*, *PAL*, *CHS*, and *CHI* were more expressed (*p* < 0.05) in the OxCM-t plants than the Wt-t and Wt-cont plants (Figure 3). This inference elucidates that the over-expression of *CM* enhances the expression of downstream genes of the phenylpropanoid pathway, which leads to the biosynthesis of secondary metabolites. Phenylalanine is the precursor for a large group of secondary metabolites, including flavonoids and lignin, and its catabolism is initiated by the phe-ammonia lyase enzyme, which is encoded by the *PAL* gene. The *PAL* gene is expressed in various plant species in response to biotic and abiotic stresses and when cell wall lignin biosynthesis is needed. Our analysis of lignin showed the same pattern to that of *CM*, *PAL*, *CHS*, and *CHI* expression (Figure 3E). Lignin biosynthesis increased with increased expression of CM and other related genes in OxCM-t plants compared with Wt-t and Wt-cont plants, indicating that lignification is enhanced during pathogen attack through transcriptional regulation of shikimate and AAA-biosynthesis-related genes. Overall, these results show that in response to WBPH attack, rice rapidly activates shikimate and phenylpropanoid pathway genes, leading to the production of lignin with a possible role in its defense mechanism.

SA is a key signaling molecule in the plant defense system against pathogenic microbes and insects. Under circumstances of *CM* over-expression in rice plants, we examined the regulation of SA, *PR1*, *PR2*, antioxidants, and their related genes to determine whether WBPH infestation activates early defense signaling compounds in association with PR genes. The accumulation of SA and expression of *PR1* and *PR2* were consistently enhanced in OxCM-t plants followed by Wt-t plants when compared with Wt-cont plants. However, the expression of *PR2* in Wt-t plants increased, but it was not significantly different from that in the Wt-cont plants (Figure 4). On the basis of the similarity of WBPH infestation to BPH, it was assumed that the basal defense system of rice was activated when the WBPHs sucked the phloem sap and secreted their saliva during infestation, which could have activated various defense signaling cascades. In association with SA, *PR* genes are also induced in infected plants, indicating that PR proteins accumulate in the infected tissues in response to WBPH, which protects plants from further infestation [18]. On the basis of function and their properties, PR proteins are divided into 17 families. Among the PR proteins, *PR1* plays an essential role in protecting plants against BPH and rice strip viruses in resistant varieties, while *PR2* is involved in the resistance of BPH in susceptible varieties [20,21]. Besides insect infestation, the expression level of the *PR1* gene is also enhanced in response to *Magnaporthe grisea* infection in rice plants [42]. Previous reports found that *PR2* was downregulated in the BPH-resistant rice plant in response to BPH infestation [21]. Our results showed that PR2 expression level was increased significantly and consistently in OxCM-t plants in response to WBPH infestation (Figure 4C). This phenomenon indicates that PR2 is upregulated in response to WBPH infestation under circumstances of *CM* over-expression. Our results are consistent with previously reported data that PR2 increases resistance to wounding and other pathogens in tobacco, carrot, and wheat plants [15]. Overall, *CM* over-expression under WBPH stress seems to increase SA accumulation and the expression of *PR1* and *PR2*.

Another mechanism of the response of rice plants to WBPHs is based on oxidative enzymes and antioxidant regulation. In the current study, we evaluated the activity of lipid peroxidase (MDA) and glutathione peroxidase (GPx) the expression of *SOD* and *CAT* (Figure 5). Lipid peroxidation is the degradation of lipids, which occurs due to the generation of ROS under conditions of stress, resulting in substantial tissue damage. However, glutathione peroxidase activity provides a detoxification mechanism for peroxides in cells, and it can protect the cells from damage by free radicals. GPx catalyzes the reduction of H_2_O_2_ and a wide range of organic peroxides to the corresponding alcohols using glutathione as a reducing agent. WBPHs generally induce strong oxidative stress in rice plant tissues and generate ROS, leading to substantial cell death and the regulation of MDA and GPx activity. In Figure 5A, histochemical staining with trypan blue indicates that substantial oxidative stress was generated in Wt-t plants compared with Wt-cont and OxCM-t plants after 6, 9, and 12 days of infestation, indicating that Wt-t plants are susceptible while OxCM-t are resistant toward WBPH. MDA enzyme activity increased significantly in Wt-t plants compared with Wt-cont (Figure 5B). However, WBPH infestation significantly increased GPx accumulation in OxCM-t plants compared with Wt-cont plants (Figure 5C). Our results validate that the reduction of MDA and enhancement of GPx activity in the transgenic line is due to the reduced stress and generation of less ROS and vice versa for Wt-t plants. The genes responsible for *SOD* and *CAT* are also strongly associated with oxidative stress. Both *SOD* and *CAT* increased significantly in OxCM-t plants in response to WBPH infestation (Figure 5D,E). Previous studies have found that *SOD, POD, PAL*, and *CAT* increased in a resistant variety after BPH infestation, which supports pathogen resistance [20,43]. Catalase, which is encoded by the *CAT* genes, is essential for mitigating oxidative stress, as it converts H_2_O_2_ into H_2_O and O_2_. The expression of the *CAT* gene is required for the detoxification of infected cells after WBPH infestation. Ding et al. [44] revealed that *SOD* plays a significant role in scavenging oxygen-free radicals, thereby avoiding oxygen-free radicals from disrupting the composition, structure, and function of cells and protecting cells from oxidative damage. *SOD* is the core of antioxidant enzymes, and it acts as the first enzyme involved in the scavenging of ROS under various environmental stresses. Therefore, we assumed that *SOD* and *CAT* expression increases in OxCM-t plants to cope with the oxidative stress caused by WBPH infestation. 

WBPH infestation inhibited the rate of photosynthesis, sugar biosynthesis, and amino acid profiling in the rice plants. Previous reports have demonstrated that phloem sap sucking pests feed on the sugar contents (sucrose, glucose and fructose) of phloem to use them as a source of energy. Initially, the chlorophyll contents were not significantly higher in OxCM-t plants than Wt-cont plants. However, due to severe infestation, there was a significant reduction in the chlorophyll of OxCM-t plants, while the Wt-t plants showed throughout reduction (Figure 6A). Unlike chlorophyll, the sugar contents were higher in the OxCM-t plants than the Wt-cont and Wt-t plants (Figure 6B). Among the amino acids, only the phenylalanine, proline, aspartic acid, and total amino acid contents increased in OxCM-t plants (Figure 6C). Severe and persistent infestation of WBPHs inhibited the rate of photosynthesis and reduced chlorophyll content. We found that after several days of infestation, the Wt-t plants turned yellowish due to the loss of chlorophyll. Previous research showed that BPH infestation reduced the chlorophyll contents in a susceptible line, but there were no changes in the chlorophyll content of a resistant line [45]. The sugar content was higher in the OxCM-t plants than Wt-cont and Wt-t plants, suggesting that the increased sugar contents in the transgenic line is due to the over-expression of the *CM* gene. However, the lack of significant difference between the sugar contents of the Wt-t and Wt-cont plants indicates that sugar is not involved in WBPH resistance. Exposure to WBPH stress leads to cellular damage due to ROS accumulation. Our results indicate that amino acids, which are strong ROS scavengers, were induced in response to WBPH infestation [46]. Among the various amino acids, proline and aspartic acid are potentially potent OH radical scavengers that can reduce oxidative stress [47,48]. 

**Figure 7:**
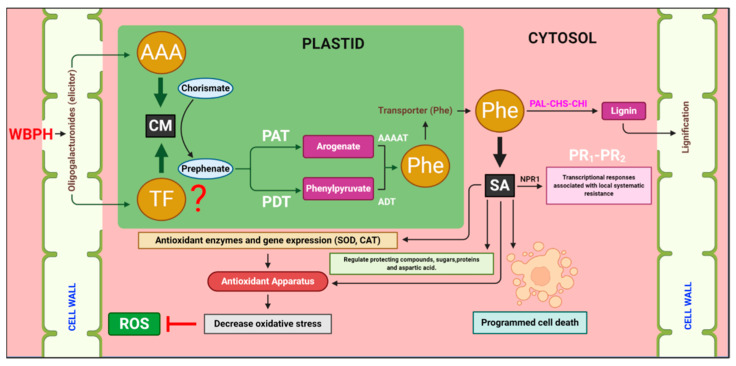
Proposed model of the molecular mechanisms of white-backed planthopper (WBPH) resistance in rice plants. WBPH infection stimulates the expression of chorismate mutase (*CM*) gene, which converts chorismate into prephenate. Prephenate is either converted into arogenate by prephenate aminotransferase (*PAT*) or to phenylpyruvate by prephenate dehydratase (*PDT*). Both arogenate and phenylpyruvate are catalyzed by aromatic amino acid amino transferase and arogenate dehydratase (*ADT*), respectively, to produce phenylalanine (Phe). This entire process occurs in the plastid. Phe is transported into the cytosol through the Phe transporter and is converted to lignin and salicylic acid. The induction of the *CM* gene regulates downstream lignin synthesizing genes, such as, phenylalanine ammonia lyase (*PAL*), chalcone synthase (*CHS*), and chalcone isomerase (*CHI*), which enhance lignification in the cell wall in response to pathogen infestation. *CM* gene induction also regulates the SA signaling, which induces catalase (MDA), glutathione peroxidase (GPx), and antioxidant-related genes, such as superoxide dismutase (*SOD*) and catalase (*CAT*), which scavenge reactive oxygen species (ROS) and reduce oxidative stress.

We propose a model of the molecular mechanisms of WBPH resistance in rice plants considering all the examined aspects of the response of rice plants to WBPHs (Figure 7). The infestation of WBPH either induced a cell wall elicitor or induced some unknown TFs, which further induced the expression of the *CM* gene. The *CM* converted chorismate into prephenate, which is the primary precursor of various aromatic amino acids, especially phenylalanine, which further initiated the secondary metabolites biosynthesis pathways. 

## 6. Conclusions

WBPH is one of the main biotic stresses and can seriously impact rice yield in several countries. Previously, conventional breeding was the main tool of selecting the best, most easily adoptable and resistant verities of crops. However, currently, molecular breeding is used to protect agriculture crops by developing new resistant varieties. Using molecular breeding techniques, we developed a highly WBPH-resistant OxF3H rice cultivar by selecting the gene of interest through QTL analysis. Plants respond to pest attack via a complex network of antioxidant, transcriptional, metabolomic and phytohormonal reprogramming. In the current study, we evaluated all possible ways of regulation of complex responses, such as the regulation of the *OsCM* gene at RNA, metabolites and hormone levels. Our results indicate that *CM* over-expression enhanced production of phenylalanine, which is the primary precursor of lignin and SA. Lignification is an essential host-oriented defense mechanism against pathogens. *CM* gene expression activates *PAL, CHS*, and *CHI* genes, which results in the biosynthesis of lignin. SA signaling pathways also have an important role in protecting the plant from WBPHs by activating PR genes and antioxidant machinery, such as *SOD* and *CAT*, which further scavenge ROS and protect the plant from oxidative stress. Overall, we conclude that WBPH induces the expression of *CM* gene, which regulates the plant defense mechanism, including the antioxidant apparatus, metabolomic induction, and signaling cascades, which enhance resistance toward WBPH infestation.

## Figures and Tables

**Table 1:** Primers designed by NCBI for qRT-PCR and accession number of selected genes.

S/No	Gene	Forward Primers	Reverse Primers	Accession No
1	*CM*	ATGGCGGCGGCGATGATTCTCTCCTGCA	TCAGGCATTGCAAGTTCGAATCCTAACAAG	XM_015793648.2
2	*PAL*	GCAACCCCAGCTTGGACTAT	TCACACTCTCGAAATGCTC	XM_015769639
3	*CHS*	AGGGAAGAATGGGGACTGAT	TGCCTCGAACTAGCATTCCT	X91811.1
4	*CHI*	AGCTCCTGAAGGCGGAAT	GATTTTCACGCGGACACC	XM_015772801
5	*PR1*	GGAAGTACGGCGAGAACATC	GGTCGTACCACTGCTTCTCC	XM_015792496.2
6	*PR2*	TGCTATGTTCGACGAGAACG	GTTGAACAGCCCAAAGTGCT	XM_015766957.2
7	*SOD*	CTTGATGCCCTGGAACCTTA	GCCAGACCCCAAAAGTGATA	KY752532.1
8	*CAT*	CCACCACAACAACCACTACGACGG	CCAACGACTCATCACACTGG	KY752529.1
9	*Actin*	ATCACCATCGGAGCAGAAAG	AAAAGATGGCTGGAAGAGCA	XM_015785964.2

**Table 2:** Glutathione peroxidase reaction scheme.

	GPx Assay Buffer (μL)	NADPH Assay Reagent (μL)	Enzyme (0.25 unit/mL) (μL)	Sample (μL)	30 mM t-Bu-OOH (μL)
Blink	940	50	---	---	10
Positive control	900	50	50	---	10
Sample	900	50	---	50	10

## Data Availability

Data is contained within the article.
